# Supplementary material for: Comparison of ultrafiltration and iron chloride flocculation in the preparation of aquatic viromes from contrasting sample types
Source: PeerJ. 2021 May 5;9:e11111. doi: 10.7717/peerj.11111 (PMC8106395; doi:10.7717/peerj.11111)
Supplement: Table S2 [file peerj-09-11111-s002.docx]

| **Date** | **Experiment** | **pH** |
| --- | --- | --- |
| 2/27/20 | Immediately After Collection | NA |
| 2/27/20 | Jar Test (Immediately after arrival to lab) | 7.98 |
| 2/28/20 | Jar Test | 8 (filtered) |
| 2/29/20 | Jar Test | 8.05 (filtered) |
| 3/2/20 | Ultrafiltration and Purification | 8 |
| 3/3/20 | Ultrafiltration and Purification | 7.91 |
| 3/4/20 | Ultrafiltration and Purification | 7.88 |
| 3/5/20 | Iron Chloride Flocculation and Purification | 7.95 |
|  | Iron Chloride Flocculation and Purification |  |
|  | Iron Chloride Flocculation and Purification |  |
