# Supplementary material for: Comparison of ultrafiltration and iron chloride flocculation in the preparation of aquatic viromes from contrasting sample types
Source: PeerJ. 2021 May 5;9:e11111. doi: 10.7717/peerj.11111 (PMC8106395; doi:10.7717/peerj.11111)
Supplement: Table S3 [file peerj-09-11111-s003.docx]

| **Phage** | **T3, T4, PhiX174** | **HS2, HM1** | **ICBM5** |
| --- | --- | --- | --- |
| **Host Media** | 8.0 g Nutrient Broth (Fisher Scientific, catalog no. BD23400), 5.0 g NaCl, 1 L H_2_O | 2.5 g Peptone, 0.5 g Yeast Extract, 100 mL Widdel 10x Salt Solution^*^, 900 mL H_2_O, pH 7.6 | 37.4 g Marine Broth 2216 (Fisher Scientific, catalog no. DF0791174), 1 mL Balch Vitamin Solutionˆ, 1 L H_2_O |
| **Hard Nutrient Agar** | 8.0 g Nutrient Broth, 5.0g NaCl, 15.0g Agar, 1 L H_2_O | 1.0 g Peptone, 0.2 g Yeast Extract, 12 g Bacto Agar, 100 mL Widdel 10x Salt Solution^*^, 900 mL H_2_O, pH 7.6 | 37.4 g Marine Broth 2216, 18 g Bacto agar, 1 mL Balch Vitamin Solutionˆ, 1 L H_2_O |
| **Soft Nutrient Agar** | 8.0 g Nutrient Broth, 5.0 g NaCl, 7.0 g Agar, 1 L H_2_O | 5 g Peptone, 1 g Yeast Extract, 6 g Bacto Agar, 100 mL Widdel 10x Salt Solution^*^, 900 mL H_2_O, pH 7.6 | 29.9 g Marine Broth 2216, 4.8 g Agar, 1 mL Balch Vitamin Solutionˆ, 1 L H_2_O |
| **Buffer** | 0.6 g NaH_2_PO_4_, 0.58 g NaCl, 0.1 g NaOH, 1 L H_2_O | 5.85 g NaCl, 20.0 g MgSO_4_⋅7H_2_O,  7.88 g Tris-HCl, 1 L H_2_O, pH 7.6 | |
| *Widdel 10x Salt Solution: 50 g NaCl, 7.5 g MgCl_2_⋅6H_2_O, 0.28 g CaCl_2_⋅2H_2_O, 0.63 g NH_4_Cl, 0.5 g KH_2_PO_4_, 1.25 g KCl, 250 mL H_2_O  ˆBalch Vitamin Solution: 25 mg para-Aminobenzoic acid, 10 mg Folic acid, 10 mg Biotin, 25 mg Nicotinic acid, 25 mg Ca pantothenate, 25 mg Riboflavin, 25 mg Thiamine hydrochloride, 50 mg Pyridoxine hydrochloride, 5 mg Cyanocobalamine, 25 mg Lipoic acid | | | |
