# Supplementary material for: Comparison of ultrafiltration and iron chloride flocculation in the preparation of aquatic viromes from contrasting sample types
Source: PeerJ. 2021 May 5;9:e11111. doi: 10.7717/peerj.11111 (PMC8106395; doi:10.7717/peerj.11111)
Supplement: Table S4 [file peerj-09-11111-s004.docx]

| **Phage** | **Centrifuge Force (*xg*)** | **Time (minutes)** |
| --- | --- | --- |
| T3 | 5000 | 15 |
| T4 | 5000 | 15 |
| PhiX174 | 1000 | 25 |
| HS2 | 3000 | 10 |
| HM1 | 3000 | 10 |
| ICBM5 | 3000 | 10 |
