# Supplementary material for: Comparison of ultrafiltration and iron chloride flocculation in the preparation of aquatic viromes from contrasting sample types
Source: PeerJ. 2021 May 5;9:e11111. doi: 10.7717/peerj.11111 (PMC8106395; doi:10.7717/peerj.11111)
Supplement: Table S5 — Resuspension buffer was diluted into sterile water (** sterile water was added instead of resuspension buffer). [file peerj-09-11111-s005.docx]

| **Iron chloride concentration (mg Fe L^-1^)** | **Resuspension buffer dilution** | **Volume of resuspension buffer added (mL)** |
| --- | --- | --- |
| 0 | NA** | 5 |
| 0.1 | 1:100 | 5 |
| 1 | 1:10 | 5 |
| 5 | 1:2 | 5 |
| 10 | 1:1 | 5 |
| 25 | 1:1 | 12.5 |
