# Supplementary material for: Comparison of ultrafiltration and iron chloride flocculation in the preparation of aquatic viromes from contrasting sample types
Source: PeerJ. 2021 May 5;9:e11111. doi: 10.7717/peerj.11111 (PMC8106395; doi:10.7717/peerj.11111)
Supplement: Table S6 — Benchtop controls are 0.5-L samples of matrix set aside and spiked with T3 or HS2 at the same time as the samples that underwent flocculation. The benchtop control remained at the same temperature as the flocculation samples throughout the experiment to assess degradation of spike viruses in each matrix over the duration of the flocculation and resuspension. The geometric mean and 95% confidence intervals are reported for all benchtop controls. [file peerj-09-11111-s006.docx]

| **Matrix** | **Recovery (%)** | |
| --- | --- | --- |
|  | **Filtrate** | **Concentrate** |
| Influent | 110.3 (59.31, 205.0) | 106.0 (54.87, 204.9) |
| Effluent | 109.2 (73.67, 161.8) | 89.50 (83.37, 96.08) |
| River Water | 81.66 (47.46, 140.5) | 132.8 (102.1, 172.7) |
| Seawater | 89.89 (54.90, 147.2) | 102.8 (54.09, 195.4) |
