# Supplementary material for: Comparison of ultrafiltration and iron chloride flocculation in the preparation of aquatic viromes from contrasting sample types
Source: PeerJ. 2021 May 5;9:e11111. doi: 10.7717/peerj.11111 (PMC8106395; doi:10.7717/peerj.11111)
Supplement: Table S7 [file peerj-09-11111-s007.docx]

| **Target Phage** | **dsDNA Fragments of qPCR Amplicons** |  |
| --- | --- | --- |
| T3 | CCA ACG AGG GTA AAG TGA TAG GCT TTA GTG TGC TTC TTG AGA CTG GTC GTT TAG TAG ACG CCA ACA ACA TCT CTC GCG CAT TGA TGG ACG AGT TCA CAT CCA ACG TTA AAG CCC ACG GTG AAG ACT TCT ACA ATG GTT GGG CCT GTC AGG TCA ACT ACA TGG AAG CGA CCC CGG ACG GCT CCC TGC GAC ACC CTA GCT TCG AGA AGT TCC GAG GAA CTG AGG ACA ACC CTC AAG AGA AAA TGT AAC CAA CTC ACT GGC TCA CCT TCA CCT TCA CGG GTG GGC CTT TCT TCG TTC CGG GCA TTA ACC CTC ACT AAC AGG AGA CAC ACA CCA TGT GGC TTA TCC TAT TCG CTA TCG TCG |  |
|  |  |  |
|  |  |  |
| HS2 | GGT TGA TGA AAA GTC ACT AGG CTG TAA ATC GCA TTC TGT AAA TAA ATC GGC ATT GTT AAG CAA TAC GCC AAT GAC TAA AGA TTC CTG CTC TAA AAT ATC CTT GTT CAT AGT TTA AAT TCC TTC ACT GCT GGT CTT GAT TGT TGT GGT TTG CCG TTA AAG CCA TTT GAT GCA GCT TTC ATT TTA GCT GAC AAG TCT GGG TAT TTA TCC CTA AGC TTT GCA AGG CTG AGA ATA TTA ACA CTC CAA AAG CTA TCA GCA TTA GCC CAT GAG AAA ACT TTC CAA CAC TCA TTT AGA TCT GCC CCG |  |
|  |  |  |
|  |  |  |
| MS2 | CCG CTA CCT TGC CCT AAA CGA AGA TCG AAA GTT TCG ATC AAA ACA CGT GGC CGG CAG GTG GTT GGA GTT GCA GTT CGG TTG GTT ACC ACT AAT GAG TGA TAT CCA GGG TGC ATA TGA GAT GCT TAC GAA GGT TCA CCT TCA AGA GTT TCT TCC TAT GAG AGC CGT ACG TCA GGT CGG TAC TAA CAT CAA GTT AGA TGG CCG TCT GTC GTA TCC AGC TGC AAA CTT CCA GAC AAC GTG CAA CAT ATC GCG ACG TAT CGT GAT ATG GTT TTA CAT AAA CGA TGC ACG TTT GGC ATG GTT GTC GTC |  |
|  |  |  |
|  |  |  |
