# Supplementary material for: Comparison of ultrafiltration and iron chloride flocculation in the preparation of aquatic viromes from contrasting sample types
Source: PeerJ. 2021 May 5;9:e11111. doi: 10.7717/peerj.11111 (PMC8106395; doi:10.7717/peerj.11111)
Supplement: Table S8 [file peerj-09-11111-s008.docx]

| **Step** | **T3** | | | | **HS2** | | | |
| --- | --- | --- | --- | --- | --- | --- | --- | --- |
|  | **Duration** | **Temperature (˚C)** | **Cycles** | **Duration** | | **Temperature (˚C)** | **Cycles** |  |
| Initial Denaturation | 2 minutes | 95 |  | 2 minutes | | 95 |  |  |
| Denaturing | 5 seconds | 95 | 35 | 5 seconds | | 95 | 35 |  |
| Annealing | 5 seconds | 60 |  | 5 seconds | | 47 |  |  |
| Extension | 25 seconds | 72 |  | 25 seconds | | 72 |  |  |
| Final Denaturation | 5 minutes | 95 | 1 | 5 minutes | | 95 | 1 |  |
| Melting Curve Initial | 15 seconds | 55 |  | 15 seconds | | 55 |  |  |
| Melting Curve End | 15 seconds | 95 |  | 15 seconds | | 95 |  |  |
| Melt Curve Duration | 20 minutes | |  | 20 minutes | | |  |  |
| Final Hold | infinite | 4 |  | infinite | | 4 |  |  |
