# Supplementary material for: Comparison of ultrafiltration and iron chloride flocculation in the preparation of aquatic viromes from contrasting sample types
Source: PeerJ. 2021 May 5;9:e11111. doi: 10.7717/peerj.11111 (PMC8106395; doi:10.7717/peerj.11111)
Supplement: Table S9 [file peerj-09-11111-s009.docx]

| **Step** | **MS2** | | |
| --- | --- | --- | --- |
|  | **Duration** | **Temperature (˚C)** | **Cycles** |
| Reverse Transcription | 15 minutes | 40 | 1 |
| Initial Denaturation | 10 minutes | 95 |  |
| Denaturing | 15 seconds | 95 | 45 |
| Annealing | 30 seconds | 60 |  |
| Extension | 45 seconds | 72 |  |
| Melting Curve Initial | 45 seconds | 68 | 1 |
| Melting Curve End | 5 seconds | 95 |  |
| Melt Curve Duration | 5 minutes | |  |
| Final Hold | infinite | 4 |  |
