# Supplementary material for: Comparison of ultrafiltration and iron chloride flocculation in the preparation of aquatic viromes from contrasting sample types
Source: PeerJ. 2021 May 5;9:e11111. doi: 10.7717/peerj.11111 (PMC8106395; doi:10.7717/peerj.11111)
Supplement: Table S10 [file peerj-09-11111-s010.docx]

| **Targets** | **Primers/Probes** | **Amplicon Length (bp)** | **Annealing Temperature (˚C)** |
| --- | --- | --- | --- |
| 16S rRNA (1) | Forward: 5’- TC CTA CGG GAG GCA GCA GT-3’  Reverse: 5’- GG ACT ACC AGG GTA TCT AAT CCT GTT-3’  Probe: 5’-/FAM/-CG TAT TAC CGC GGC TGC TGG CAC-/BHQ_1/-3’ | 466 bp | 56 |
| T3 | Forward: 5’- CCA ACG AGG GTA AAG TGA TAG-3’  Reverse: 5’- CGA CGA TAG CGA ATA GGA TAA G-3’  Probe: 5’-/HEX/-CC AAC AAC ATC TCT CGC GCA TT-/BHQ_2/-3’ | 351 bp | 56 |
| T4 (2) | Forward: 5’-CCA CAA CTA ACC GAG GAA GTA A-3’  Reverse: 5’-TGC GAT ATG CTA TGG GTC TTG-3’  Probe: 5’-/FAM/-TGC TCC ATC AGA GGA AGA ATG CGA-/BHQ_1/-3’ | 107 bp | 56 |
| PhiX174 | Forward: 5’-GGG ATA CCC TCG CTT TCC TG-3’  Reverse: 5’-CAA AGA CGA GCG CCT TTA CG-3’  Probe: 5’-/HEX/-TAC GTG CGG AAG GAG TGA TGT AAT G-/BHQ_2/-3’ | 353 bp | 56 |
| HS2 | Forward: 5’-GGT TGA TGA AAA GTC ACT-3’  Reverse: 5’-CGG GGC AGA TCT AAA TGA-3’  Probe: 5’-/HEX/-TTT AGT CAT TGG CGT ATT GCT TAA C-/BHQ_2/-3’ | 300 bp | 57 |
| HM1 | Forward: 5’-CGT CTG CAG TAG ATT GGG CA-3’  Reverse: 5’-AGA TGG GGT GTT GGA GGA AAG-3’  Probe: 5’-/FAM/-CAA GAA CAG GAC TTG CCA GAA GTG T-/BHQ_1/-3’ | 216 bp | 56 |
| ICMB5 | Forward: 5’-ATC CGA TCC GCC GAA GTA AC-3’  Reverse: 5’-AAA CGC TCC GTT CTT CTC GT-3’  Probe: 5’-/HEX/-AAG GTG TAA CCG CTG GTC GGC ATA A-/BHQ_2/-3’ | 275 bp | 56 |

References

1. Nadkarni MA, Martin FE, Jacques NA, Hunter N. 2002. Determination of bacterial load by real-time PCR using a broad-range (universal) probe and primers set. Microbiology 148:257-266.

2. Lim SW, Lance ST, Stedman KM, Abate AR. 2017. PCR-activated cell sorting as a general, cultivation-free method for high-throughput identification and enrichment of virus hosts. Journal of Virological Methods 242:14-21.
