# Supplementary material for: Comparison of ultrafiltration and iron chloride flocculation in the preparation of aquatic viromes from contrasting sample types
Source: PeerJ. 2021 May 5;9:e11111. doi: 10.7717/peerj.11111 (PMC8106395; doi:10.7717/peerj.11111)
Supplement: Table S11 — The initial denaturation step was only used on dsDNA targets (ssDNA targets were PhiX174 and ICBM5). With ssDNA phage targets, the initial denaturing began to degrade the ssDNA targets and produced two positive fluorescence levels. *Annealing temperatures for each target are provided in Table S10. [file peerj-09-11111-s011.docx]

| **Step** | **Temperature (˚C)** | **Duration (minutes)** | **Cycles** |
| --- | --- | --- | --- |
| Initial Denaturation (dsDNA targets only) | 95 | 10 | 1 |
| Denaturation | 95 | 0.5 | 40 |
| Annealing | * | 1 |  |
| Extension | 72 | 2 |  |
| Final Annealing | 4 | 5 | 1 |
| Final Denaturation | 95 | 5 | 1 |
| Hold | 4 | Infinite | 1 |
