# Supplementary material for: Comparison of ultrafiltration and iron chloride flocculation in the preparation of aquatic viromes from contrasting sample types
Source: PeerJ. 2021 May 5;9:e11111. doi: 10.7717/peerj.11111 (PMC8106395; doi:10.7717/peerj.11111)
Supplement: Table S16 — The geometric mean and geometric standard deviation from the triplicate data is provided. Individual t-tests were performed for each matrix and phage spike. [file peerj-09-11111-s016.docx]

| **Matrix** | **Phage Spike** | **Virus Recovery (%)** | | | | **Virus to 16S rRNA Enrichment** | | | |
| --- | --- | --- | --- | --- | --- | --- | --- | --- | --- |
|  |  | **Ultrafiltration** | **Flocculation** | **p-values** | **Ultrafiltration** | | **Flocculation** | **p-values** |  |
| Influent | T3 | 47 (24, 93) | 25 (16, 38) | 0.066 (ns) | 3600 (1500, 8600) | | 1000 (680, 44) | 0.049 (*) |  |
| Secondary Effluent | T3 | 42 (37, 49) | 21 (4.7, 99) | 0.21 (ns) | 70 (32, 150) | | 44 (26, 75) | 0.97 (ns) |  |
|  | T4 | 17 (9.6, 30) | NA | NA | 54 (50, 58) | | NA | NA |  |
|  | PhiX174 | 3.8 (2.7, 5.3) | NA | NA | 12 (9.4, 16) | | NA | NA |  |
| River Water | T3 | 43 (39, 47) | 25 (6.6, 92) | 0.11 (ns) | 440 (310, 640) | | 19 (5.3, 71) | 8.0E-3 (**) |  |
| Seawater | HS2 | 18 (5.3, 58) | 15 (13, 16) | 0.47 (ns) | 94 (48, 180) | | 8.8 (2.6, 29) | 0.034 (*) |  |
|  | HM1 | 30 (7.9, 110) | 28 (23, 34) | 0.68 (ns) | 160 (79, 310) | | 17 (4.5, 64) | 0.034 (*) |  |
|  | ICBM5 | 13 (5.7, 28) | 2.2 (1.4, 3.5) | 0.042 (*) | 68 (29, 160) | | 1.3 (0.54, 3.2) | 0.041 (*) |  |
