# Supplementary material for: Comparison of ultrafiltration and iron chloride flocculation in the preparation of aquatic viromes from contrasting sample types
Source: PeerJ. 2021 May 5;9:e11111. doi: 10.7717/peerj.11111 (PMC8106395; doi:10.7717/peerj.11111)
Supplement: Supplemental Information 17 — Three methods to purify viral nucleic acids were tested on pre-concentrated influent and secondary effluent samples. Each purification method tested has a different mechanism for purifying the viral nucleic acids. Two chloroform treatments, chloroform and DNase treatments, and filtering followed by DNase treatment were evaluated by the ratio of phage T3 gene copies to 16S rRNA gene copies with a higher ratio indicating a better purification performance. For the influent samples, the highest phage T3 to 16S rRNA ratios are observed in the chloroform and DNase treated samples. The phage T3 to 16S rRNA ratio is not significantly less than the two chloroform treated samples (p-value = 0.28) in the secondary effluent as seen in Figure S1. The chloroform and DNase treatment method was selected for purifying viruses in all of the sample matrices for the iron chloride flocculation and purification and ultrafiltration and purification methods. [file peerj-09-11111-s017.docx]

**Section S1: Optimizing Purification Methods**

Three methods to purify viral nucleic acids were tested on pre-concentrated influent and secondary effluent samples. Each purification method tested has a different mechanism for purifying the viral nucleic acids. Two chloroform treatments, chloroform and DNase treatments, and filtering followed by DNase treatment were evaluated by the ratio of phage T3 gene copies to 16S rRNA gene copies with a higher ratio indicating a better purification performance. For the influent samples, the highest phage T3 to 16S rRNA ratios are observed in the chloroform and DNase treated samples. The phage T3 to 16S rRNA ratio is not significantly less than the two chloroform treated samples (*p*-value = 0.28) in the secondary effluent as seen in Figure S1. The chloroform and DNase treatment method was selected for purifying viruses in all of the sample matrices for the iron chloride flocculation and purification and ultrafiltration and purification methods.

***Methods.*** Three different methods to purify viruses were tested: two chloroform treatments, a chloroform and DNase treatment, and filtering and DNase treatment. The methods were tested in triplicate on a concentrated influent and concentrated secondary effluent. 10-L of influent and 20-L of secondary effluent were collected the day before the experiment and concentrated approximately 30-fold and 65-fold, respectively, by tangential ultrafiltration with the same method described above then 0.45-µm Express PLUS filtered. Samples were stored at 4˚C overnight. The day of the experiment, the wastewater samples were concentrated an additional 20-fold by dead-end ultrafiltration using 100 kDa MWCO and 1 cm^2^ surface area Amicon^TM^ filter units. Initially, 500 µL of sample was added to each filter, then centrifuged at 3,000*xg* and 4˚C until approximately 200 µL remained. The process continued with more sample added to each filter until a total of 4 mL of sample was added and a 200 µL final volume remained on each filter. The concentrate was collected by inverting the filter into a clean collection tube and centrifuging at 1,000*xg* for 1 minute. Approximately 10^6^ T3 gene copies µL^-1^ were added to the influent and secondary effluent after concentrating. A sample was collected for recovery analysis after T3 addition and stored at 4˚C until DNA extraction. Each replicate had an initial volume of 400 µL.

Chloroform treatments were performed by adding 100 µL of chloroform and vortexing for approximately 2 minutes then settled for 10 minutes and centrifuged briefly. A majority of the chloroform was removed by pipetting the chloroform off of the bottom of the samples, then evaporating the remainder from the sample by aerating the sample in a fume hood. The chloroform treatment was completed by filtering samples through 0.45-µm PES and 13-mm diameter syringe filters (CellTreat Scientific Supplies, Cat. No. 229748). For the two chloroform treatments samples, the entire chloroform treatment protocol was repeated. Filtering for the filtering and DNase treatment samples was performed with the same syringe filters as the final step of the chloroform treatment. DNase treatment was performed as described for iron chloride flocculation and purification methods. Samples were collected for recovery analysis after purification and stored at 4˚C until DNA extraction. Previously described T3 and 16S rRNA probe ddPCR assays were used in the recovery analysis.
