# Supplementary material for: Comparison of ultrafiltration and iron chloride flocculation in the preparation of aquatic viromes from contrasting sample types
Source: PeerJ. 2021 May 5;9:e11111. doi: 10.7717/peerj.11111 (PMC8106395; doi:10.7717/peerj.11111)
Supplement: Figure S1 — The geometric mean surrounded by individual points is plotted. A 2-way ANOVA test was performed to investigate variability in the data. No statistically significant differences between the three treatments in the influent were observed (all p-values > 0.25). No statistical difference between two chloroform treatments and chloroform and DNase treatments in secondary effluent was observed (p-value = 0.30). A statistical difference between two chloroform treatments and filtering and DNase treatments in secondary effluent was observed (p-value = 0.04). Chloroform and DNase treatments and filtering and DNase treatments in secondary effluent had no statistically significant difference (p-value = 0.45). [file peerj-09-11111-s018.pdf]

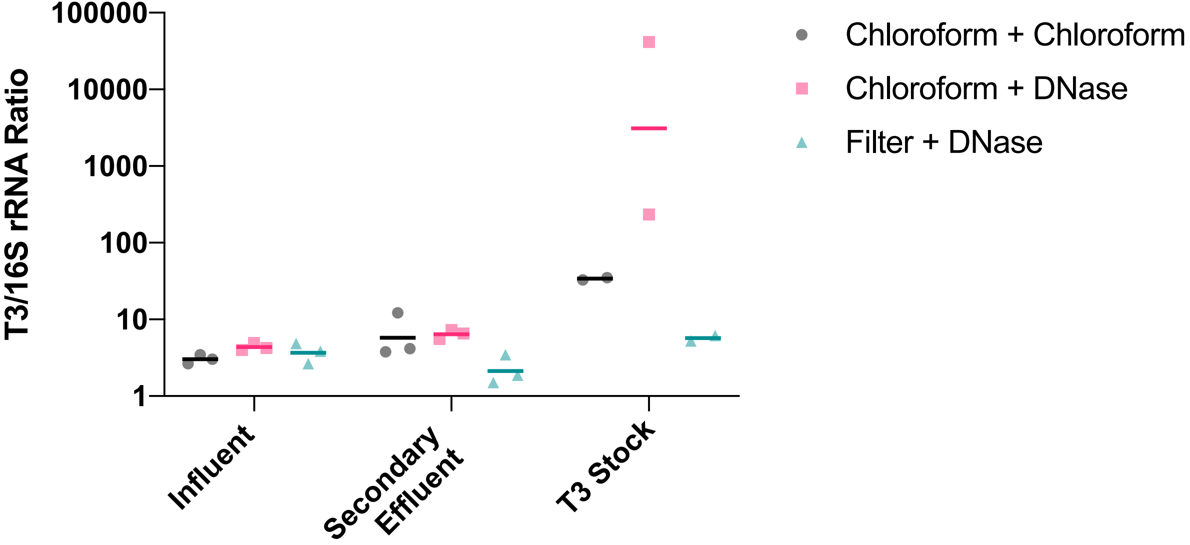

T3/16S rRNA Ratio
100000
10000
1000
100
10
1
Influent
Secondary Effluent
T3 Stock
Chloroform + Chloroform
Chloroform + DNase
Filter + DNase
