# Supplementary material for: Comparison of ultrafiltration and iron chloride flocculation in the preparation of aquatic viromes from contrasting sample types
Source: PeerJ. 2021 May 5;9:e11111. doi: 10.7717/peerj.11111 (PMC8106395; doi:10.7717/peerj.11111)
Supplement: Supplemental Information 19 — MilliQ pre-wash, 1% BSA incubation, and sonication have been previously reported to improve viral recoveries after dead-end ultrafiltration. We tested these three methods alone and in combination with our samples spiked with T3 and MS2 to confirm previous findings. We found no significant difference in the geometric mean of any of the treatment recoveries or the no treatment control (one-way ANOVA: p-value = 0.23). We did not perform any additional treatments before or after dead-end ultrafiltration for the remainder of the experiments. [file peerj-09-11111-s019.docx]

**Section S2: Optimizing Dead-End Ultrafiltration**

MilliQ pre-wash, 1% BSA incubation, and sonication have been previously reported to improve viral recoveries after dead-end ultrafiltration (1). We tested these three methods alone and in combination with our samples spiked with T3 and MS2 to confirm previous findings. We found no significant difference in the geometric mean of any of the treatment recoveries or the no treatment control (one-way ANOVA: *p*-value = 0.23). We did not perform any additional treatments before or after dead-end ultrafiltration for the remainder of the experiments.

***Methods.*** Three different methods to optimize dead-end ultrafiltration were tested: milliQ pre-wash, bovine serum albumin (BSA) treatment pre-ultrafiltration, and sonication post-ultrafiltration. These treatments were all tested in triplicate separately and in combination against a no treatment control. The experiment was conducted with secondary effluent collected the day of the experiment and concentrated approximately 70-fold by tangential ultrafiltration then 0.45-µm Express PLUS filtered. Tangential ultrafiltration was performed as described for the ultrafiltration and purification method. Approximately 10^5^ gene copies µL^-1^ T3 and 10^8^ gene copies µL^-1^*Escherichia coli* phage MS2 (ATCC^®^ 15597-B1^TM^) were added to the concentrated effluent. A sample was collected after the spike addition for recovery analysis and stored at 4˚C until DNA extraction. The milliQ pre-wash was conducted by adding 500 µL of sterilized milliQ to each filter then centrifuging at 3,000*xg* and 4˚C until all the milliQ passed through the filter. The milliQ was 0.02-µm filtered with Anotop^TM^ 25 Plus sterile syringe filters (GC Healthcare Whatman^TM^, Cat. No. 0992626) to sterilize. The BSA treatment was performed with a sterile 1% BSA solution made by diluting 50 mg mL^-1^ UltraPure BSA (Invitrogen^TM^, Cat. No. AM2616) in phosphate buffer (5 mM NaH_2_PO_4_ and 10 mM NaCl, pH 7.5). 500 µL of the 1% BSA solution was added to each filter and incubated at room temperature for 1 h, then removed from the filter by pipetting. Dead-end ultrafiltration was performed with 100 kDa MWCO and 1 cm^2^ surface area Amicon^TM^ Ultra Centrifugal filter units by adding 500 µL of concentrated effluent to each filter and centrifuging at 3,000*xg* and 4˚C until 100 µL of sample remains on the filters. Filters were sonicated by adding 50 µL of sterile 1x TE buffer and sonicating for 3 minutes at 50 W and 42 kHz. All filters were inverted into clean collection tubes and centrifuged at 1000*xg* and 4˚C for 1 minute. After treatments, samples were collected for recovery analysis and stored at 4˚C until DNA extraction. T3 qPCR and MS2 RT-qPCR assays were used in the recovery analysis.

***Phage MS2 RT-qPCR assay.*** Primer (5’ to 3’) specific to phage MS2 were selected (303 bp; forward, CCG CTA CCT TGC CCT AAA C; reverse, GAC GAC AAC CAT GCC AAA C)^2^. The 20 µL reaction contained 10 µL 2x GoTaq^TM^ one-step RT-qPCR master mix (Promega, Cat. No. PRA6020), 0.4 µL 50x reverse transcriptase mix, 0.3 µM primers, and 2 µL of DNA template. Standard curves were prepared in triplicate between 100 and 10^6^ gene copies µL^-1^ with gBlocks dsDNA fragments of the amplicon sequence (IDT, Coralville, IA) shown in Table S7. qPCR was performed on a realplex^2^ Mastercycler epgradient S automated real-time PCR system (Eppendorf^®^, New York City, New York) with reaction conditions are provided in Table S9. All efficiencies were greater than 80% and R^2^ values were greater than 0.95. Each sample was measured in duplicate and the geometric mean was taken.

1. Deng L, Gregory A, Yilmaz S, Poulos BT, Hugenholtz P, Sullivan MB. 2012. Contrasting life strategies of viruses that infect photo- and heterotrophic bacteria, as revealed by viral tagging. mBio 3.
