# Supplementary figures and images for: Comparison of ultrafiltration and iron chloride flocculation in the preparation of aquatic viromes from contrasting sample types
Source: PeerJ. 2021 May 5;9:e11111. doi: 10.7717/peerj.11111 (PMC8106395; doi:10.7717/peerj.11111)

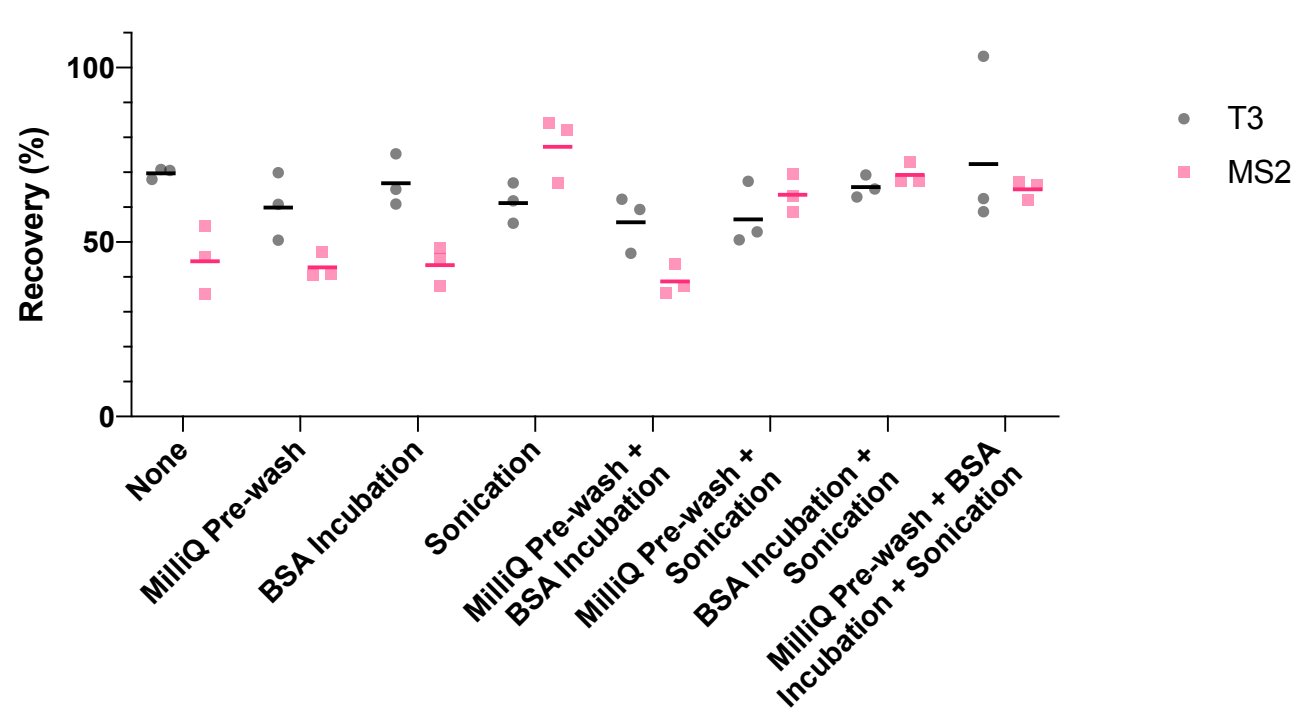

Supplement: Figure S2 — The secondary effluent was previously tangentially ultrafiltered (60-fold concentration) and 0.45-µm filtered. T3 and MS2 were spiked in to evaluate the recovery after dead-end ultrafiltration (5-fold concentration) for each method. The experiment was performed in triplicate. The individual measurements and geometric mean for each virus and dead-end ultrafiltration condition are shown above. A one-way ANOVA test concluded there is no statistical difference between the means of the different dead-end ultrafiltration methods (p-value = 0.23). [file peerj-09-11111-s020.pdf]

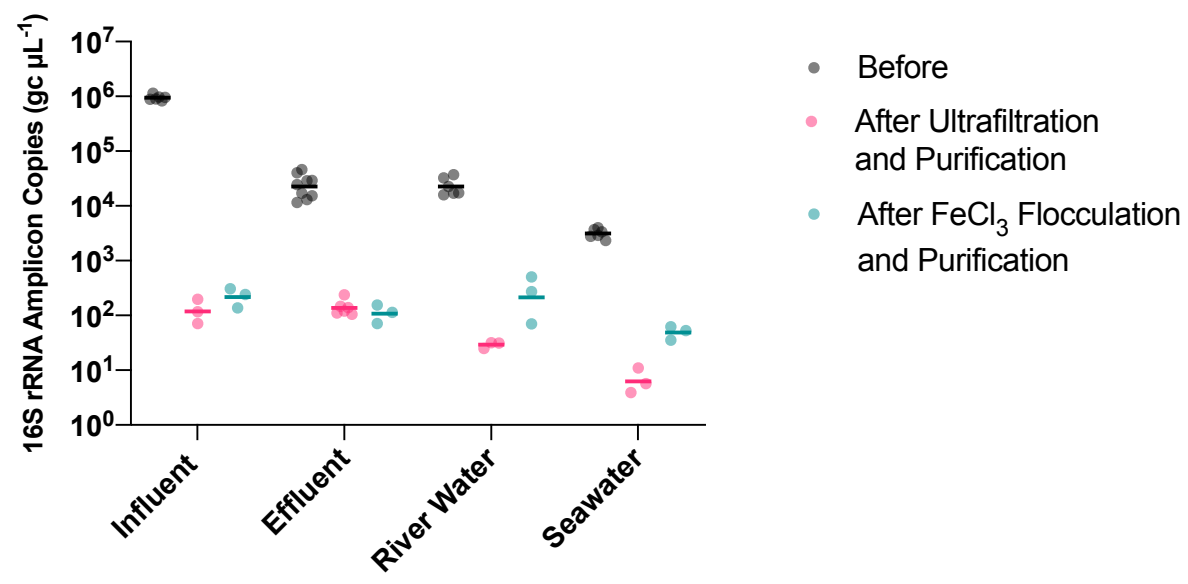

Supplement: Figure S3 — The geometric mean and individual points are plotted for each matrix and treatment. [file peerj-09-11111-s022.pdf]

**A****Ultrafiltration**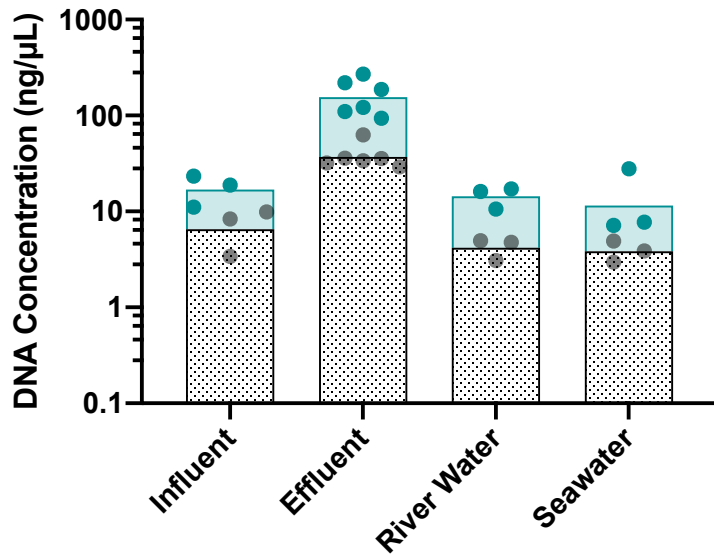**B****Iron Chloride Flocculation**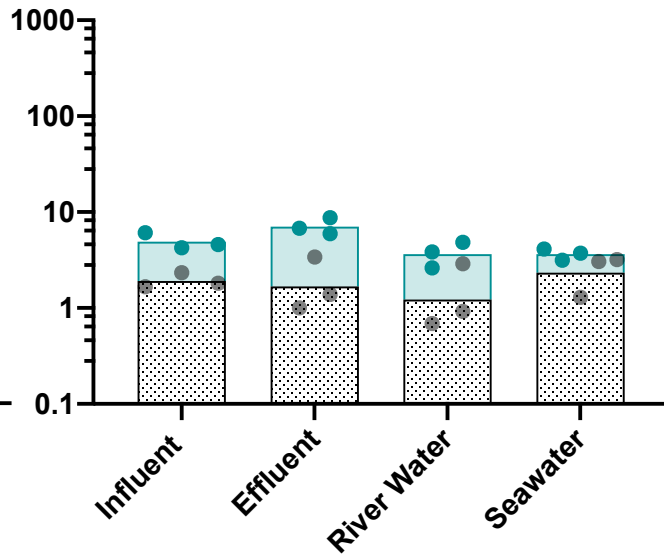

● Total DNA    ● dsDNA

Supplement: Figure S4 — DNA concentrations after ultrafiltration and purification (A) and iron chloride flocculation and purification (B). Geometric means of ssDNA concentrations with individual experimental replicates are stacked on top of geometric means of dsDNA concentrations with individual experimental replicates. [file peerj-09-11111-s023.pdf]

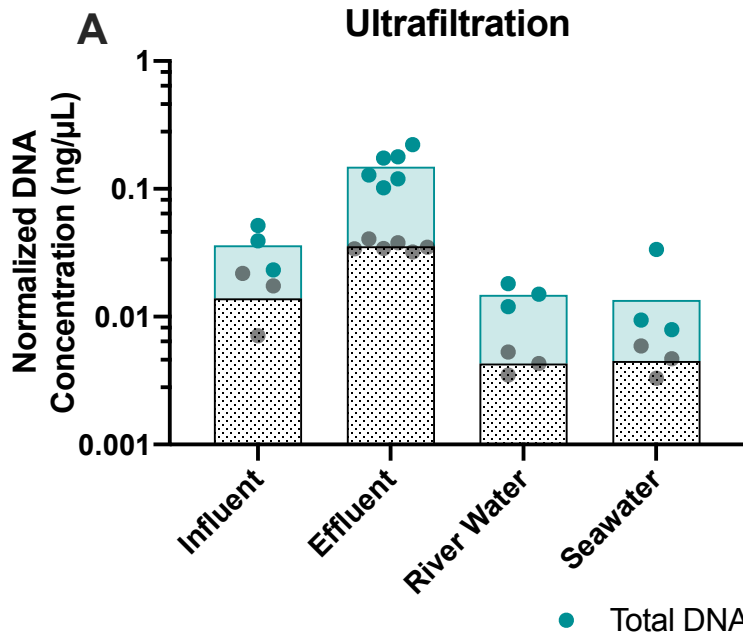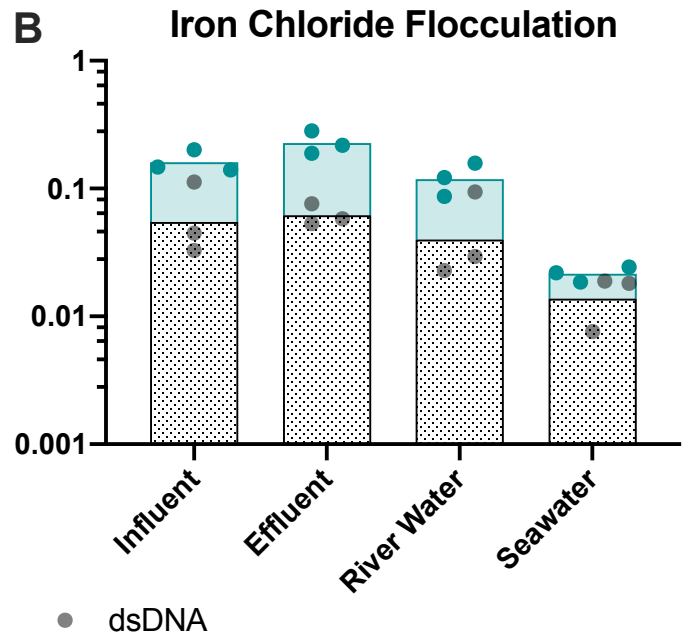

Supplement: Figure S6 — Normalized DNA concentrations by the fold concentration factor for each individual sample after ultrafiltration and purification (A) and iron chloride flocculation and purification (B). Geometric means of normalized ssDNA concentrations with individual experimental replicates are stacked on top of geometric means of normalized dsDNA concentrations with individual experimental replicates. [file peerj-09-11111-s025.pdf]
