# Supplementary material for: Comparison of ultrafiltration and iron chloride flocculation in the preparation of aquatic viromes from contrasting sample types
Source: PeerJ. 2021 May 5;9:e11111. doi: 10.7717/peerj.11111 (PMC8106395; doi:10.7717/peerj.11111)
Supplement: Supplemental Information 21 — After two hours of incubation at room temperature and DNase treatment, 2.1% and 2.9% of marine phage HS2 was recovered in two 0.45-µm filtered influent samples. This indicates that differences in seawater and wastewater matrices impact the survivability of HS2. Based on this result, marine phages (HS2, HM1, ICBM5) were spiked into seawater and freshwater phages (T3, T4, PhiX174) were spiked into freshwater matrices for all experiments. [file peerj-09-11111-s021.docx]

**Section S3: Phage HS2 Survivability in Wastewater Influent**

After two hours of incubation at room temperature and DNase treatment, 2.1% and 2.9% of marine phage HS2 was recovered in two 0.45-µm filtered influent samples. This indicates that differences in seawater and wastewater matrices impact the survivability of HS2. Based on this result, marine phages (HS2, HM1, ICBM5) were spiked into seawater and freshwater phages (T3, T4, PhiX174) were spiked into freshwater matrices for all experiments.

***Methods.*** Two 25 mL of influent was 0.45-µm PES filtered and spiked with 10^6^ gc µL^-1^ of HS2. The samples incubated on the benchtop for 2 hours, then were treated with DNase, as described previously, with a 2 hour incubation time. DNase treatment was performed to remove non-encapsidated HS2 genomes and constrain qPCR measurements to HS2 viral particles. DNA extractions were performed before, immediately after HS2 addition, and after incubation and DNase treatment, as described previously and HS2 recovery was determined based on measurements from the HS2 qPCR assay.
