# Supplementary material for: Comparison of ultrafiltration and iron chloride flocculation in the preparation of aquatic viromes from contrasting sample types
Source: PeerJ. 2021 May 5;9:e11111. doi: 10.7717/peerj.11111 (PMC8106395; doi:10.7717/peerj.11111)
Supplement: Figure S5 — DNA fragmentation for each matrix and method (triplicates pooled, 9 total samples) was assessed by Agilent TapeStation for DNA lengths up to 60,000 bp by the Advanced Genomics Core at the University of Michigan. The clearly defined bands in the ultrafiltration samples are relics of the T3 spikes in freshwater matrices and HS2 spike in seawater that notably do not appear in the iron chloride flocculation samples. This observation indicates potential genome shearing during the iron chloride flocculation and purification process that may not occur in the ultrafiltration and purification process. [file peerj-09-11111-s024.pdf]

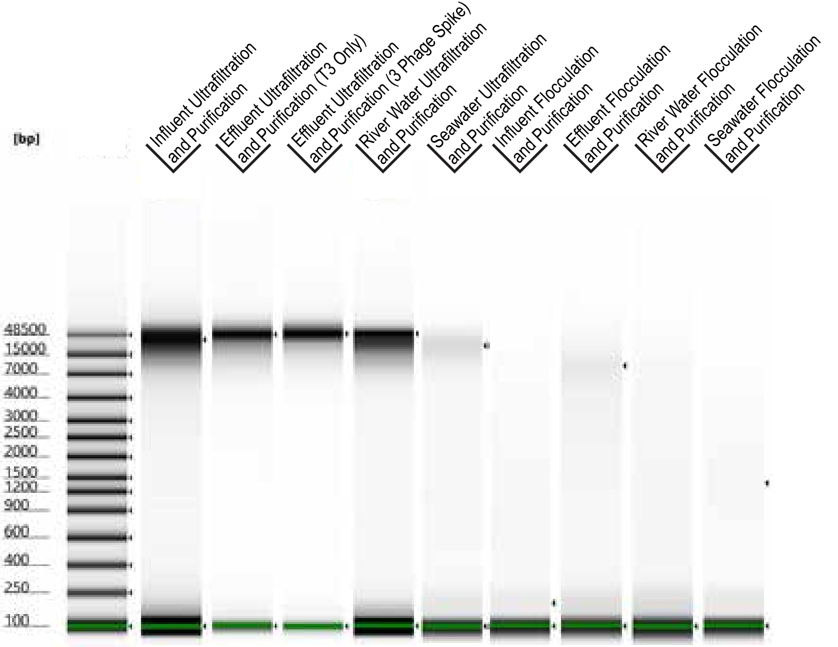
[bp]
48500
15000
7000
4000
3000
2500
2000
1500
1200
900
600
400
250
100
Influent Ultrafiltration and Purification
Effluent Ultrafiltration and Purification (T3 Only)
Effluent Ultrafiltration and Purification (3 Phage Spike)
River Water Ultrafiltration and Purification
Seawater Ultrafiltration and Purification
Influent Flocculation and Purification
Effluent Flocculation and Purification
River Water Flocculation and Purification
Seawater Flocculation and Purification
